# Polyphenolic Composition, Antioxidant and Antibacterial Activities for Two Romanian Subspecies of *Achillea distans* Waldst. et Kit. ex Willd

**DOI:** 10.3390/molecules18088725

**Published:** 2013-07-24

**Authors:** Daniela Benedec, Laurian Vlase, Ilioara Oniga, Augustin C. Mot, Grigore Damian, Daniela Hanganu, Mihaela Duma, Radu Silaghi-Dumitrescu

**Affiliations:** 1 Department of Pharmacognosy “Iuliu Hatieganu” University of Medicine and Pharmacy, 12 I. Creanga Street, Cluj-Napoca 400010, Romania; E-Mails: dbenedec@umfcluj.ro (D.B.); ioniga@umfcluj.ro (I.O.); 2 Department of Pharmaceutical Technology and Biopharmaceutics, 12 I. Creanga Street, Cluj-Napoca 400010, Romania; E-Mail: laurian.vlase@umfcluj.ro; 3 Department of Chemistry and Chemical Engineering ”Babes-Bolyai”University, 11 A. Janos Street, Cluj-Napoca 400028, Romania; E-Mails: augustinmot@chem.ubbcluj.ro (A.C.M.); rsilaghi@chem.ubbcluj.ro (R.S.-D.); 4 Department of Physics, ”Babes-Bolyai”University, 11 A. Janos Street, Cluj-Napoca 400028, Romania; E-Mail: grigore.damian@phys.ubbcluj.ro; 5 State Veterinary Laboratory for Animal Health and Safety, 1 Piata Marasti Street, Cluj-Napoca 400609, Romania; E-Mail: duma.alexandru-cj@ansvsa.ro

**Keywords:** *Achillea distans*, polyphenols, antioxidant activity, hemoglobin, EPR

## Abstract

The aim of this work was to study the chemical composition, antioxidant and antibacterial properties of *Achillea distans* Waldst. et Kit. subsp. *distans* and *Achillea distans* Waldst. et Kit. subsp. *alpina* Rochel, from the Rodna Mountains (Romania). The identification and quantification of major phenolic compounds was performed by a HPLC-MS method. The total polyphenolic and flavonoid content was determined spectrophotometrically. The antioxidant activity was evaluated using the DPPH bleaching method, trolox equivalent antioxidant capacity assay (TEAC), hemoglobin ascorbate peroxidase activity inhibition (HAPX) assay, and an Electron Paramagnetic Resonance (EPR) spectroscopy method. A data indicated that *A. distans* subsp. *alpina* extract has more antioxidant activity than *A. distans* subsp. *distans* extract. Luteolin, apigenin, quercetin, caffeic and chlorogenic acids were present in the two extracts of *A. distans*, but in different amounts. Three flavonoids were detected only in *A. distans* subsp. *alpina*. The polyphenol-richer *A. distans* subsp. *alpina* extract showed a higher antioxidant activity than *A.* *distans* subsp. *distans* extract. *A.* *distans* subsp. *distans* extract showed inhibitory activity for Gram-positive bacteria, as evaluated with four species. The quantitative and qualitative differences between the two subspecies of *Achillea distans* could be used as a potential taxonomic marker in order to distinguish the species.

## 1. Introduction

The genus *Achillea* (*Asteraceae*) is represented by about 85 species throughout the World, and 23 species and 10 varieties or subspecies can be found in the Romanian flora. Some *Achillea* species have ethnopharmacologic importance and are known to be used in folk remedies for various purposes [1,2]. *Achillea distans* Waldst. et Kit. ex Willd. (Alps yarrow) is an Alpino-Carpatho-Balkan species that vegetates on the upper limit of mountain forests and in subalpine shrubs. According to the length of ligulate florets and to their color, two subspecies are recognized: *Achillea distans* Waldst. et Kit. subsp. *Distans*, with white flowers and about 2 mm length of ligulae and *Achillea distans* Waldst. et Kit. subsp. *alpina* (Rochel) Soó, with pink flowers and about 3 mm length of ligulae [1,2,3]. Due to the differences in the chemical composition of the essential oils of *A. distans* subsp. *distans* and *A*. *distans* subsp. *alpine*, these were considered as infraspecific chemical taxa or chemovarieties of *Achillea distans* [3]. *Achillea distans* Waldst. et Kit. ex Willd., found in the Rodna Mountains (a subdivision of the Eastern Carpathians in Northern Romania), was confirmed as a native species of the Romanian flora [1]. The flower heads of *A. distans* species contain essential oils, polyphenolic compounds, terpenoids; the essential oil and the tincture showed antimicrobial and anti-inflammatory activities [4,5,6,7]. The roots of *A. distans* subsp. *distans* also contain essential oil [8].

Phenolic compounds are a major group of compounds acting as primary antioxidants or free radical scavengers; flavonoids are a ubiquitous group of the polyphenolic substances which are present in all higher plants [9]. The total phenolic compounds and flavonoid content are also related to the antioxidant activity. Over the past decade, major advances have been made to investigate the antioxidant and antimicrobial properties of different *Achillea* species: *A. alexandri-regis* Bornm. & Rudsky [10], *A. biebersteinii* Afan. [11], A. *moschata* Wulf., *A. distans* Waldst. & Kit. ex Willed. [12], *A. micrantha* Willd., *A. filipendula* Lam., *A. tenuifolia* Lam., *A. vermicularis* Trin., and *A. wilhelmsii* C. Koch. [13], *A. santolina* [14], *A. ptarmica* L., *A. nobilis* L. [15], *A. millefolium* L. [13,16,17,18,19], *A. ligusta* All. [20], *A. collina* Becker, *A. pannonica* Scheele [21,22], *A. teretifolia* Waldst. and Kitt, *A. schischkinii* Sosn. [23]. Only one of the studies evaluated the antioxidant activity of *Achillea distans* from northern Italy, showing that methanolic extracts from the flowering parts possess antioxidant activity, by employing DPPH and LDL oxidation assays [12]. Scientific data on the two subspecies (*alpina* and *distans*) of *A. distans* are very limited.

The aim of the present paper was to characterize the polyphenolic composition of hydroalcoholic extracts from the flowers of two subspecies of *Achillea distans* Waldst. et Kit. ex Willd.: *Achillea distans* Waldst. et Kit. subsp. *distans* and *Achillea distans* Waldst. et Kit. subsp. *alpina* (Rochel) Soó, and to evaluate their *in vitro* antioxidant and antibacterial activities.

## 2. Results and Discussion

### 2.1. HPLC Analysis of Polyphenols

**Figure 1:**
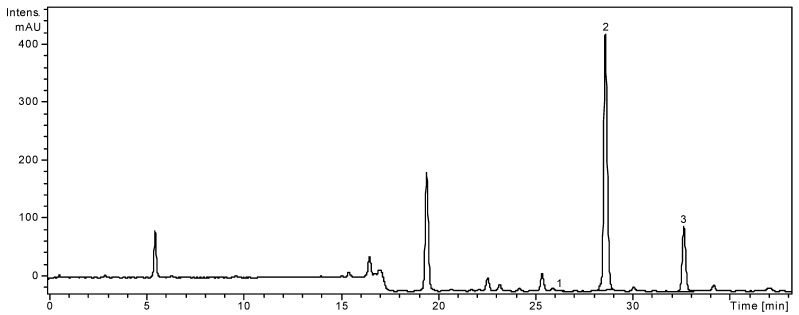
HPLC chromatogram of *A. distans* subsp. *distans.*

*Notes*: Chromatographic conditions were as given in the Experimental section. The identified compounds: 1, Quercetin;2, Luteolin 3, Apigenin.

**Figure 2:**
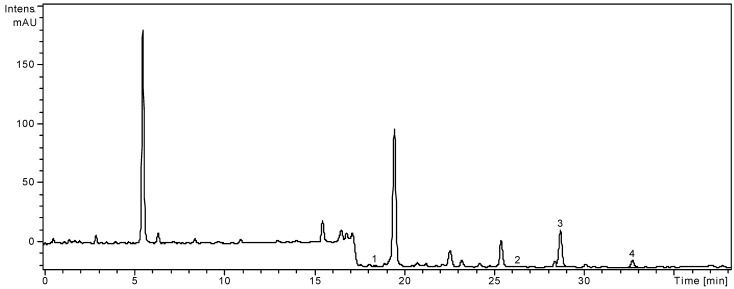
HPLC chromatogram of *A. distans* subsp. *alpina.*

*Notes*: Chromatographic conditions were as given in the Experimental section. The identified compounds: 1, Hyperoside; 2, Quercetin; 3, Luteolin; 4, Apigenin.

HPLC coupled with MS is a very powerful analytical technique, due to its high sensitivity and the structural information that can be obtained about the analytes. A high-performance liquid chromatographic (HPLC) method has been developed for the determination of 19 phenolic compounds (eight phenolic acids, four quercetin glycosides, and seven flavonol and flavone aglycones) from plant material. The applicability of the proposed analytical method and the qualitative and quantitative determination of the standard phenolic compounds have already been verified [4,24,25,26]. The method allows a simultaneous analysis of different classes of polyphenols by a single pass column (the separation of all examined compounds was carried out in 35 min). The concentrations of identified polyphenolic compounds in both analyzed samples are presented in Table 1. They were shown in the order of their retention time. The HPLC chromatogram of *A. distans* subsp. *distans* sample is presented in Figure 1 and the HPLC chromatogram of *A. distans* subsp. *alpina* sample is presented in Figure 2. The quantitative determination was performed using the external standard method (Table 2).

In the ethanolic extract of *A. distans* subsp. *distans* flowers, luteolin was the compound found in the largest amount (763.12 ± 1.88 mg/100 g) followed by apigenin (264.84 ± 1.16 mg/100 g). We detected quercetin at lower levels than major flavonoides (1.44 ± 0.06 mg/100 g). Caffeic acid and chlorogenic acid were also identified in this extract, but they were in too low concentration to be quantified (Table 1).

**Table 1:** Polyphenolic compounds content in *Achillea distans* subspecies (mg/100 g plant material).

Polyphenolic compounds	*m/z* value	tR ± SD (min)	*Achillea distans* subsp. *distans*	*Achillea distans* subsp. *alpina*
Caffeic acid	179	5.60 ± 0.04	<0.2	<0.2
Chlorogenic acid	353	5.62 ± 0.05	<0.2	<0.2
Hyperoside	463	18.60 ± 0.12	NF	2.05 ± 0.10
Isoquercitrin	463	19.60 ± 0.10	NF	<0.2
Rutin	609	20.20 ± 0.15	NF	<0.2
Quercetin	301	26.80 ± 0.15	1.44 ± 0.06	1.11 ± 0.09
Luteolin	285	29.10 ± 0.19	763.12 ± 1.88	52.65 ± 0.85
Apigenin	279	33.10 ± 0.15	264.84 ± 1.16	13.47 ± 0.53

Note: NF - not found, below limit of detection. Values are the mean ± SD (n = 3).

**Table 2:** Retention times (RT) of polyphenolic compounds (min).

Peak no.	Phenolic compounds	*m/z*	t_R_ ± SD (min)
1.	Caftaric acid	311	3.54 ± 0.05
2.	Gentisic acid	179	6.52 ± 0.04
3.	Caffeic acid	179	5.60 ± 0.04
4.	Chlorogenic acid	353	5.62 ± 0.05
5.	*p*-Coumaric acid	163	9.48 ± 0.08
6.	Ferulic acid	193	12.8 ± 0.10
7.	Sinapic acid	223	15.00 ± 0.10
8.	Cichoric acid	473	15.96 ± 0.13
9.	Hyperoside	463	18.60 ± 0.12
10.	Isoquercitrin	463	19.60 ± 0.10
11.	Rutin	609	20.20 ± 0.15
12.	Myricetin	317	21.13 ± 0.12
13.	Fisetin	285	22.91 ± 0.15
14.	Quercitrin	447	23.64 ± 0.13
15.	Quercetin	301	26.80 ± 0.15
16.	Patuletin	331	29.41 ± 0.12
17.	Luteolin	285	29.10 ± 0.19
18.	Kaempferol	285	32.48 ± 0.17
19.	Apigenin	279	33.10 ± 0.15

*Note*: SD, standard deviation.

In the extract of *A. distans* subsp. *alpina* flowers, the two free aglycones, *i.e.*, apigenin and luteolin, were found in much smaller amounts than in “*distans*” subspecies (13.47 ± 0.27, and 52.65 ± 0.85 mg/100 g, respectively). Quercetin was detected in small amounts (1.11 ± 0.09 mg/100 g). In this extract, three flavonoid glycosides were identified. Hyperoside (quercetin 3-*O*-galactoside) was determined in quantities of 2.05 ± 0.10 mg/100 g plant material. Rutin (quercetin 3-*O*-rutinoside) and isoquercitrin (quercetin 3-*O*-glucoside) were identified in the ethanolic extract, but they were in too low concentration to be quantified. The three glycosides of quercetin detected only in *A. distans* subsp. *alpina*, could be used as a potential taxonomic marker in order to distinguish the subspecies. Caffeic acid and chlorogenic acid were also identified in the extract of *A. distans* subsp. *alpina* (Table 1). Considering the 19 standard compounds used in this study (Table 2), some other peaks were not identified.

Thus, the comparative study showed large differences, especially quantitative, between the two taxa of *Achillea distans*. The richest subspecies in aglycones of flavonoids (luteolin, apigenin, quercetin) was *A. distans* subsp. *distans*, but the flavonoid glycosides were not found.

### 2.2. Polyphenolic and Flavonoid Contents of the Extracts

Flavonoids and phenolic acids make up one of the most pervasive groups of plant phenolics. The plants and herbs consumed by humans may contain various amounts of different phenolic acid and flavonoid components. The effect of dietary phenolics is currently of great interest due to their antioxidant and possible anticarcinogenic activities. Phenolic acids and flavonoids also function as reducing agents, free radical scavengers, and quenchers of singlet oxygen. In addition, flavonoids and phenolic acids components play important roles in the control of cancer and other human diseases. Due to their importance in plants and human health, it would be useful to know the concentration of the polyphenolic compounds and biological activities that could indicate their potentials as therapeutic agents, and also for predicting and controlling the quality of medicinal herbs [27].

The total polyphenolic content (TPC) values summarized for *Achillea* extracts in Table 3 were quantified based on the linear equation obtained from gallic acid standard calibration curve. Thus, TPC values were expressed as gallic acid equivalent (mg GAE/g sample). The calculation of total flavonoid content of plant extracts was carried out using the standard curve of rutin and presented as rutin equivalents (mg RE/g sample). The highest amount of the total polyphenols was determined in the extract of *A. distans* subsp. *alpina* flowers (174.75 ± 1.47 mg·g^−1^) followed by *A. distans* subsp. *distans* extract (101.61 ± 1.24 mg·g^−1^). Concerning the content of flavonoids, contrary, the extract of *A. distans* subsp. *distans* (37.26 ± 0.71 mg·g^−1^) was richer in flavonoids, than the extract of *A. distans* subsp. *alpina* (33.18 ± 0.60 mg·g^−1^).

**Table 3:** The content of total polyphenols and flavonoids in *A. distans extracts.*

Samples	TPC (mg GAE/g plant material)	Flavonoids (mg RE/g plant material)
*A. distans* subsp. *distans*	101.61 ± 1.24	37.26 ± 0.71
*A. distans* subsp. *alpina*	174.75 ± 1.47	33.18 ± 0.60

Each value is the mean ± SD of three independent measurements. GAE: Gallic acid equivalents; RE: rutin equivalents.

### 2.3. Antioxidant Activity

The antioxidant capacity of the ethanolic extracts of flowers heads of *A. distans* subsp. *distans* and *A. distans* subsp. *alpina* was determined by several methods: DPPH bleaching assay, the Trolox equivalent antioxidant capacity (TEAC), Electron Paramagnetic Resonance (EPR) method, and the hemoglobin ascorbate peroxidase activity inhibition (HAPX) assay (Table 4).

**Table 4:** Antioxidant capacity parameters obtained using several methods for studied *Achillea* samples.

Samples	DPPH (% decolorization)	IC_50_ (µg·mL^−1^)	TEAC (µmol Trolox/mg plant material)	HAPX (%)
*A. distans* subsp. *distans*	52.73 ± 0.77	204.85 ± 2.93	42.14 ± 0.48	4.91 ± 1.24
*A. distans* subsp. *alpina*	93 ± 1.47	83.80 ± 1.20	46.72 ± 0.35	10.27 ± 1.81
Quercetin	-	5.60 ± 0.35	-	-
BHT	-	16 ± 0.54	-	-

Each value is the mean ± SD of three independent measurements.

**Figure 3:**
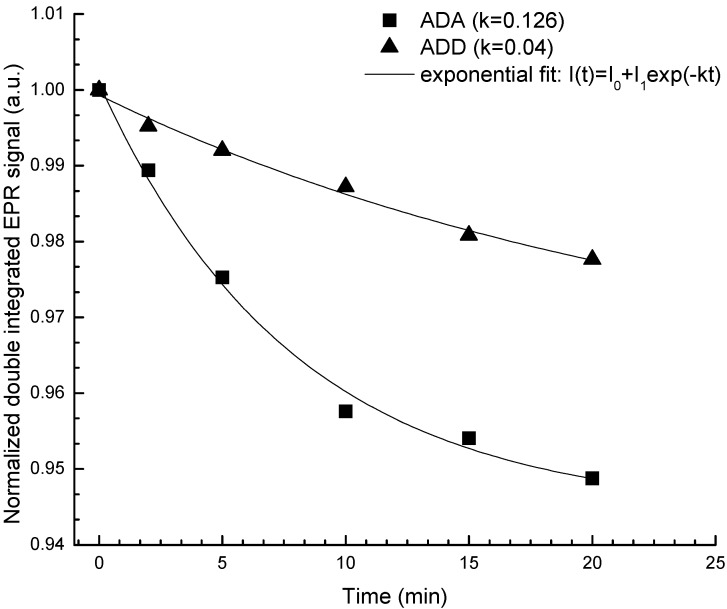
TEMPO consumption (1.4 mM) by *A. distans* subsp. *distans* (ADD) and *A. distans* subsp. *alpina* (ADA). The experimental data were fitted with exponential decay function according to the equation I(t) = I_0_ + I_1_e^−kt^, and the kinetic constants are k_ADA_ = 0.126; k_ADD_ = 0.04. Gallic acid was used as control, k_gallic acid_ = 0.131, at a concentration of 6 mg/mL.

The antioxidant activity of the ethanol extracts was further assessed by the DPPH radical bleaching method. The DPPH scavenging ability of the subspecies *alpina* was 1.76 times larger than that of the subspecies *distans* at the same concentration (218.75 µg plant product/mL extract) (Table 4). The highest radical scavenging activity was showed by the extract of *A. distans* subsp. *alpina* with IC_50_ = 83.80 ± 1.20 µg·mL^−1^, followed by the extract of *A. distans* subsp. *distans* (IC_50_ = 204.85 ± 2.93). This is in good agreement with the TPC values listed in Table 3. Compared to the reference compounds, quercetin (IC_50_ = 5.60 ± 0.35 µg·mL^−1^) and BHT (IC_50_ = 16 ± 0.54 µg·mL^−1^), the ethanol extracts of *A. distans* showed lower antioxidant capacity. According to this method, *A. distans* subsp. *alpina* extract exhibited a moderate antioxidant capacity (50 µg/mL < IC_50_ ≤ 100 µg·mL^−1^), and the ethanol extract of *A.* *distans* subsp. *distans* has no relevant antioxidant activity (IC_50_ > 200 µg·mL^−1^). In the TEAC assay, the stable radical is dissolved in an aqueous solution, thus expecting a different mechanism of interaction between antioxidants molecule and the radical since TEAC assesses the more hydrophilic components while DPPH describes all components. This can be observed by the final TEAC values (Table 4), even though the antioxidant power is significantly higher in *A. distans* subsp. *alpina* extract, the difference in absolute value is not so great as in the case of DPPH method. Thus, it can be speculated that the *A. distans* subsp. *distans* extract contain antioxidant compounds which better act in aqueous solution than in organic solvent (ethanol). In the HAPX assay one measures, in a physiological relevant manner, the capability of the extract components to quench the HbFe^IV^ resulted by hydrogen peroxide-induced damage upon HbFe^III^. The results further indicate that by this assay too, *A. distans* subsp. *alpina* extract is double more antioxidant than *A. distans* subsp. *distans* extract. Figure 3 illustrates the results of an antioxidant assay using electron paramagnetic spectroscopy (EPR). This assay also indicate that *A. distans* subsp. *alpina* extract has a comparable activity to that of gallic acid and *A. distans* subsp. *distans* extract has three times less activity than gallic acid. *A. distans* subsp. *alpina* extract is much more antioxidant than *A. distans* subsp. *distans* extract.

### 2.4. Antibacterial Activity

The antibacterial activity of the ethanol extracts of *A. distans* is shown in the Table 4. The antibacterial activity is ranked from no activity (-: inhibition diameter < 10 mm), low (+: inhibition diameter between 10 and 15 mm), moderate (++: inhibition diameter between 15 and 20 mm) and high activity (+++: diameter inhibition ≥ 20 mm). The extracts were investigated for their *in vitro* antimicrobial properties using a disk-diffusion method against *S. aureus*, *L. monocytogenes*, *E. coli*, *S. typhymurium* [28,29] (Table 5).

**Table 5:** Antibacterial activity (inhibition zone expressed in mm) ^a^ of two investigated extracts of *A. distans*.

Samples	Inhibition zone in diameter (mm)			
	*Staphylococcus aureus*	*Listeria monocytogenes*	*Escherichia coli*	*Salmonella typhymurium*
*A. distans* subsp. *distans*	13.0 ± 0.05	17.0 ± 0.1	10.0 ± 0.05	7.0 ± 0.00
*A. distans* subsp. *alpina*	12.0 ± 0.07	12.0 ± 0.00	8.0 ± 0.05	6.0 ± 0.02
Gentamicin	19 ± 0.05	18 ± 0.1	22 ± 0.00	18 ± 0.05

*Notes*: ^a^ The values represent the average of three determinations ± standard deviations. Gentamicin (10 µg/disk) was used as a positive control.

The extract of *A. distans* subsp. *distans* showed a moderate activity towards *L. monocytogenes* (inhibition diameter between 15 and 20 mm), a low antibacterial effect towards *S. aureus* and *E. coli*(inhibition diameter between 10 and 15 mm), and inactive against S. *typhymurium* (inhibition diameter < 10 mm). The extract of *A. distans* subsp. *alpina* showed a low antibacterial activity towards *S. aureus*, *L. monocytogenes*, and it did not show antibacterial activity against the tested gram-negative bacterial strains. 

## 3. Experimental

### 3.1. Plant Material and Extraction Procedure

The flowers of *Achillea distans* Waldst. et Kit. ex Willd. subsp. *distans* (voucher No. 953) and the flowers of *Achillea distans* Waldst. et Kit. subsp. *alpina* (Rochel) Soo (voucher No.954) were collected in August 2011 (Rodna Mountains, in the north of the Eastern Carpathians, near Iezer Lake, at 1700–1750 m altitude, Romania) in the blossom period. Voucher specimens were deposited in the Herbarium of the Department of Pharmaceutical Botany of the Faculty of Pharmacy, Cluj-Napoca, Romania. The plant material (flowers) was reduced to a proper degree of fineness. 2.0 g of the material was extracted with 20 mL of 70% ethanol (Merck, Darmstadt, Germany), for 30 min on a water bath, at 60 °C. The samples were then cooled down and centrifuged at 4,500 rpm for 15 min, and the supernatant was recovered [24,26].

### 3.2. Chemicals

Chlorogenic acid, *p*-coumaric acid, caffeic acid, rutin, apigenin, quercetin, isoquercitrin, quercitrin, hyperoside, kaempferol, myricetol, fisetin from Sigma (St. Louis, MO, USA), ferulic acid, sinapic acid, gentisic acid, gallic acid, patuletin, luteolin from Roth (Karlsruhe, Germany), cichoric acid, caftaric acid from Dalton (Toronto, ON, Canada). HPLC grade methanol, analytical grade orthophosphoric acid, hydrochloric acid and Folin-Ciocalteu reagent were purchased from Merck, hydrogen peroxide, ABTS (2,2′-azinobis-3-ethylbenzotiazoline-6-sulphonic acid) were from Sigma (St. Louis, MO, USA); aluminum chloride, sodium acetate, sodium carbonate, ethanol (Merck), DPPH (2,2-diphenyl-1-picrylhydrazyl) and BHT (butylated hydroxytoluene) were obtained from Alfa-Aesar (Karlruhe, Germany). Bovine hemoglobin was purified following the general protocol of Antonini and Brunori [30]. The met forms of hemoglobin were prepared by ferricyanide treatment as previously described [31]. All microorganism products were distributed by MicroBioLogics®: *Staphylococcus aureus* ATCC 49444 (Gram+), *Listeria monocytogenes* ATCC 13076 (Gram+), *Escherichia* *coli* ATCC 25922 (Gram-), and *Salmonella typhymurium* ATCC 14028 (Gram-).

### 3.3. HPLC/MS Analysis

#### 3.3.1. Apparatus and Chromatographic Conditions

The experiment was carried out using an Agilent Technologies 1100 HPLC Series system (Agilent, Santa Clara, CA, USA) equipped with G1322A degasser, G13311A binary gradient pump, column thermostat, G1313A autosampler and G1316A UV detector. The HPLC system was coupled with an Agilent 1100 mass spectrometer (LC/MSD Ion Trap VL). For the separation, a reverse-phase analytical column was employed (Zorbax SB-C18 100 × 3.0 mm i.d., 3.5 μm particle); the work temperature was 48 °C. The detection of the compounds was performed on both UV and MS mode. The UV detector was set at 330 nm until 17.5 min, then at 370 nm. The MS system operated using an electrospray ion source in negative mode. The chromatographic data were processed using ChemStation and DataAnalysis software from Agilent. The mobile phase was a binary gradient: methanol and acetic acid 0.1% (v/v). The elution started with a linear gradient, beginning with 5% methanol and ending at 42% methanol, for 35 min; then 42% methanol for the next 3 minutes [24,25,26]. The flow rate was 1 mL·min^−1^ and the injection volume was 5 µL.

The MS signal was used only for qualitative analysis based on specific mass spectra of each polyphenol. The MS spectra obtained from a standard solution of polyphenols were integrated in a mass spectra library. Later, the MS traces/spectra of the analysed samples were compared to spectra from library, which allows positive identification of compounds, based on spectral mach. The UV trace was used for quantification of identified compounds from MS detection. Using the chromatographic conditions described above, the polyphenols eluted in less than 40 min (Table 2). Four polyphenols cannot be quantified in current chromatographic conditions due overlapping (caftaric acid with gentisic acid and caffeic acid with chlorogenic acid). However, all four compounds can be selectively identified in MS detection (qualitative analysis) based on differences between their molecular mass and MS spectra. For all compounds, the limit of quantification was 0.5 μg/mL, and the limit of detection was 0.1 μg·mL^−1^. The detection limits were calculated as minimal concentration producing a reproductive peak with a signal-to-noise ratio greater than three. Quantitative determinations were performed using an external standard method. Calibration curves in the 0.5–50 μg·mL^−1^ range with good linearity (R^2^ > 0.999) for a five point plot were used to determine the concentration of polyphenols in plant samples [24,25,26].

#### 3.3.2. Identification and Quantification of Polyphenols

The detection and quantification of polyphenols was performed in UV assisted by mass spectrometry detection. Due to peak overlapping, four polyphenol-carboxylic acids (caftaric, gentisic, caffeic, chlorogenic) were determined only based on MS spectra, whereas for the rest of the compounds the linearity of the calibration curves was very good (R^2^ > 0.998), with detection limits in the range of 18 to 92 ng·mL^−1^. The detection limits were calculated as the minimal concentration yielding a reproducible peak with a signal-to-noise ratio greater than three. Quantitative determinations were performed using an external standard method; retention times were determined with a standard deviation ranging from 0.04 to 0.19 min (Table 2). For all compounds, the accuracy was between 94.13% and 105.3%. Accuracy was checked by spiking samples with a solution containing each polyphenol in a 10 μg·mL^−1^ concentration. In all analyzed samples the compounds were identified by comparison of their retention times and recorded electrospray mass spectra with those of standards in the same chromatographic conditions. 

### 3.4. Determination of Total Polyphenols and Flavonoids Content

The total phenolic content (TPC) of the extracts was determined by the Folin-Ciocalteau method with some modifications [32,33,34,35]. Each ethanolic extract (2 mL) diluted 25 times was mixed with Folin-Ciocalteu reagent (1.0 mL) and distilled water (10.0 mL) and diluted to 25.0 mL with a 290 g/L solution of sodium carbonate. The samples were incubated in the dark for 30 min. The absorbance was measured at 760 nm. Gallic acid was used as standard for the calibration curve and was plotted at 0.02, 0.04, 0.06, 0.08, and 0.10 mg·mL^−1^, prepared in methanol-water (50:50, v/v). TPC values were determined using an equation obtained from the calibration curve of gallic acid graph (R^2^ = 0.9990).

The spectrophotometric aluminum chloride method was used for flavonoids determination. Each extract (5 mL) was mixed with sodium acetate (5.0 mL, 100 g·L^−1^), aluminum chloride (3.0 mL, 25 g·L^−1^, and filled up to 25 mL by methanol in a calibrated flask. The absorbance was measured at 430 nm [36]. Total flavonoids content values was determined using an equation obtained from calibration curve of the rutin graph (R^2^ = 0.9996).

### 3.5. Antioxidant Activity Test

#### 3.5.1. DPPH• Radical Scavenging Assay

The free radical scavenging activity of the ethanolic extracts of the two subspecies of *A. distans* was measured in terms of hydrogen donating or radical scavenging ability using the stable DPPH radical method. A DPPH solution (0.1 g·L^−1^) in methanol was prepared and this solution (4.0 mL) was added to of extract solution (or standard) in methanol at different concentrations ((4.0 mL, 10–50 μg·mL^−1^). After 30 min of incubation at 40 °C in a thermostatic bath, the decrease in the absorbance (n = 3) was measured at 517 nm. The percent DPPH scavenging ability was calculated as: DPPH scavenging ability = (A_control_ − A _sample_/A_control_) × 100, where Abs_control_ is the absorbance of DPPH radical + methanol (containing all reagents except the sample) and Abs_sample_ is the absorbance of DPPH radical + sample extract. Afterwards, a curve of % DPPH scavenging capacity *versus* concentration was plotted and IC_50_ values were calculated. IC_50_ denotes the concentration of sample required to scavenge 50% of DPPH free radicals [16,21,34,37,38,39,40]. The positive controls were those using the standard solution quercetin and butylated hydroxytoluene (BHT). IC_50_ value is related with the antioxidant capacity. So, IC_50_ ≤ 50 µg/mL value means a high antioxidant capacity; 50 µg/mL < IC_50_ ≤ 100 µg·mL^−1^ value means a moderate antioxidant capacity and IC_50_ > 200 µg·mL^−1^ value means no relevant antioxidant capacity [39]. 

#### 3.5.2. Trolox Equivalent Antioxidant Capacity (TEAC) Assay

In a quartz cuvette, to phosphate buffer saline (PBS, 955 μL) the following were added: *Achillea* extracts (20 μL, diluted 100 times), and ABTS^+●^ (25 μL, from 74 mM stock solution). The experiments were done in duplicates, with a relative standard deviation of less than 6%. The *Achillea* extract in the assay mixture was 8.24 mg·L^−1^. The content of the generated ABTS^●+^ radical was measured at 734 nm after 600 s reaction time and was converted in Trolox equivalents by the use of a calibration curve (R^2^ = 0.9987) constructed with 0, 2, 4, 6, 8, 10 mg·L^−1^ Trolox standards [41,42].

#### 3.5.3. Hemoglobin/Ascorbate Peroxidase Activity Inhibition (HAPX) Assay

In a quartz cuvette sodium acetate buffer (956 μL, 50 mM, pH 5.5), was mixed with ascorbic acid (7 μL, 50 mM), hydrogen peroxide (20 μL, 50 mM) and 10-times diluted extracts of *A. distans* ssp. *distans* and *A. distans* ssp. *alpina* (10 μL, final concentration of 82.4 mg L^−1^). After 12–15 s, met-hemoglobin (met-Hb) from a 1.4 mM stock solution (7 μL) was added to the reaction mixture and the 290 nm absorbance was further monitored. A measurable significant inhibition of the ascorbic acid consumption was observed compared to the reference (run in four different experiments) in which the extract was replaced by an equal amount of extraction solvent [31,43]. The slope of each sample was calculated at the tested concentration and also without the tried sample (blank). The inhibition of the ascorbic acid consumption was determined as follows: HAPX = 100 − [(slope of the sample/slope of the blank) × 100]. All the spectroscopic measurements were performed using a Jasco V-530 UV-Vis spectrophotometer (Jasco International Co., Ltd., Tokyo, Japan).

#### 3.5.4. EPR (Electron Paramagnetic Resonance) Spectroscopy Method

The EPR spectra were measured using an EMX Micro spectrometer (Bruker BioSpin GmbH, Rheinstetten, Germany). EPR instrument conditions were as follows: microwave frequency 9.43 GHz, microwave power 15.89 mW, modulation frequency 100 kHz, modulation amplitude 3 G, sweep rate 10 G/s; time constant 10.24 ms, average of three sweeps for each spectrum, room temperature. For the 2,2,6,6-tetramethylpiperidin-1-yl)oxyl (TEMPO) radical scavenging by the extract monitored by EPR, 3.43 mM TEMPO (20 µL) was quickly mixed with extract (30 µL) and transferred with a syringe to an an EPR micro tube. The EPR signal is registered at defined time interval and the double integrals are calculated. The kinetic profile obtained is fitted with a first order exponential decay function and the kinetic constant is considered an antioxidant parameter [44]. Gallic acid was used as control.

### 3.6. Antibacterial Activity Test

The ethanolic extracts of *A. distans* were tested for antimicrobial activity against two Gram-positive bacterial strains: *Staphylococcus aureus* (ATCC 49444), *Listeria monocytogenes* (ATCC 13076), and against two Gram-negative bacterial strains: *Escherichia coli* (ATCC 25922), *Salmonella typhymurium* (ATCC 14028) by a previously described disc diffusion method, in Petri dishes [28,29]. Each microorganism was suspended in Mueller Hinton (MH) broth and diluted approximately to 10E6 colony forming unit (cfu)/mL. They were “flood-inoculated” onto the surface of MH agar and MH Dextroxe Agar (MDA) and then dried. Six-millimeter diameter wells were cut from the agar using a sterile cork-borer, and 60 μL of each extract were delivered into the wells. The plates were incubated at 37 °C and the diameters of the growth inhibition zones were measured after 24 h. Gentamicin (10 μg/well) was used as positive control. The controls were performed with only sterile broth and with only overnight culture and 10 μL of 70% ethanol [28,29]. All tests were performed in triplicate, and clear halos greater than 10 mm were considered as positive results.

### 3.7. Statistical Analysis

All the samples were analyzed in triplicate, except those for EPR method wich were analyzed in duplicate; the average and the relative SD were calculated using the Excel software package. 

## 4. Conclusions

We have determined the phenolic profile, the antioxidant and antibacterial activities for two indigenous subspecies of *Achillea distans* and we have completed the literature data with new information concerning the polyphenolic compounds and their bioactivity. The simultaneous determination of a wide range of polyphenolic compounds was performed using a rapid, highly accurate and sensitive HPLC method assisted by mass spectrometry detection. The antioxidant activity evaluated using the DPPH (2,2-diphenyl-1-picrylhydrazyl) bleaching method, Trolox equivalent antioxidant capacity assay (TEAC), hemoglobin ascorbate peroxidase activity inhibition (HAPX) assay, and an Electron Paramagnetic Resonance (EPR) spectroscopy method, indicate that *A. distans* subsp. *alpina* extract is more antioxidant than *A.* *distans* subsp. *distans* extract, related with the polyphenolic total content.

The comparative study showed significant differences, both qualitative and especially quantitative, between the two taxa of *Achillea distans.* This study suggests that the flowers of *A*. *distans* subsp. *alpina* and *A. distans* subsp. *distans* from Romania may be considered a source of important polyphenols with bioactive properties, a source that could be pharmaceutically exploited.
